# Migrants’ experiences as health ambassadors during the COVID-19 pandemic in Norway-a qualitative study

**DOI:** 10.1186/s12939-025-02480-9

**Published:** 2025-04-30

**Authors:** Marta-Johanne Svendsen, David Lackland Sam, Oddvar Kaarboe, Esperanza Diaz

**Affiliations:** 1 Pandemic Centre, Department for Global Public Health and Primary Care, University of Bergen, Bergen, 5009 Norway; 2 Department of Psychosocial Science, University of Bergen, Bergen, 5020 Norway; 3 Department of Health and Functioning, Western Norway University of Applied Sciences, Bergen, 5063 Norway

**Keywords:** Pandemic, Migrants, Stress, Resilience, Coping

## Abstract

**Background:**

In response to the high incidence of COVID-19 infections among migrants, and lack of information, several interventions relying on migrants to deliver information to their peers were implemented. Although these strategies seem to be effective, the experiences of the migrants disseminating health information have not been studied. The Health Ambassador Project (HA-project) was one of such projects connecting key members from migrant communities (Health Ambassadors (HAs)) with health experts and the municipality, to disseminate health information to migrants. The HA-project was conducted in Bergen, Norway, in 2021–2022. This study aims to understand the experiences of the HAs during their involvement in the HA-project, how their role affected them in positive or negative ways and how they coped with eventual difficulties in the role.

**Methods:**

We conducted individual interviews in January 2023 with seven HAs. The interviews were transcribed and analyzed using systematic text condensation.

**Findings:**

Participating in the HA-project was predominantly a positive experience for all HAs, by acquiring a sense of meaning and of being considered a valuable resource during the COVID-19 pandemic. There were positive social and professional aspects related to being able to meet other HAs and learning from each other’s experiences. However, psychological distress was reported related to the responsibility of conveying correct health information about the COVID-19 pandemic to fellow migrants and fighting misinformation. Several HAs used more of their free time than was expected of them, helping people in practical ways. HAs from Ukraine joined the project in 2022 and experienced their roles as more stressful given limitations in the availability of interpretation when collaborating with the experts.

**Conclusion:**

Targeted health interventions relying on migrants to address their peers should consider the experiences of those distributing information and minimize their psychological stress.

**Supplementary Information:**

The online version contains supplementary material available at 10.1186/s12939-025-02480-9.

## Introduction

The overrepresentation of vulnerable populations among the infected and the deceased during the COVID-19 pandemic was a reminder of the necessity of equity in health for resilient health care responses [1]. To counteract this unequal development in terms of burden of disease, several interventions to improve public health through diversity sensitive approaches were launched in a number of countries, often in collaboration with the most severely affected communities. Now that the pandemic is over, it is imperative to learn from the experiences of those involved in these interventions to prepare for fairer responses to health crises in the future [2].

Internationally, migrants were from an early stage overrepresented among the infected and admitted to hospital due to the SARS-CoV-2 virus [3–8], and health authorities globally understood that the pandemic could not be fought with pockets of ongoing infection [9]. The solution in several countries was to engage the migrant communities themselves to convey adapted information in their own language to their members. Understanding the effect of the spokesperson’s contribution on public health message sharing [10], information brokers were typically selected as volunteer migrants with key roles in their communities. In this way, much of the responsibility of reaching out with health information was placed in the hands of the voluntary sector, typically religious communities or non-governmental organizations (NGOs).

### Strategies of communication in Norway

Similar patterns of infections among migrants and health authorities´responses took place in Norway [5], where some migrant groups had more than twice infection rates and risk of dying because of the virus as compared to individuals born in Norway [9, 11–13]. To ameliorate the situation, it soon became clear that communication between health authorities and the migrants had to be improved through co-produced targeted interventions that the communities could rely on (14–15). To this end, the Directorate of Integration and Diversity (IMDi) granted funding to projects in collaboration with civil society, aiming to reach different migrant groups with timely and practical information about the pandemic. Information should be given in the community´s own language and through trusted channels [16]. Most of the information brokers were selected on the basis of being key persons in their respective communities and their demonstrated willingness to contribute. In a minority of the projects, however, the volunteers had some health-related background, even if they were not necessarily working as professional health workers. Although these strategies of communication with migrants through key community persons were later evaluated as useful to decrease infection rates [17], the experiences of the migrants working as information brokers to their peers have, to our knowledge, not been studied. However, the new roles assumed by these individuals acting as information brokers may have been challenging due to a lack of relevant qualifications, scarce access to reliable and understandable information, continually changing information about rules and norms, limited resources to distribute information themselves and their personal situation as members of the same community in the midst of a health crisis [5, 15, 18].

### Psychological and practical effects

In the general population, volunteers and health care workers experienced higher levels of psychological distress compared to the rest of the population during the pandemic [19]. Despite this, both groups reported higher levels of happiness than the rest of the population [19]. However, we are unaware how migrants working as bridges between cultures and languages experienced their roles during the COVID-19 pandemic, or how they coped with eventual challenges-that is, the cognitive and behavioral strategies they employed to navigate challenging situations [20]. 

The HA-project was one of the projects financed by IMDi [16]. To close this knowledge gap, in this paper we explore how migrants who participated as key-informants in the HA-project were affected through their new roles disseminating health information to their local communities during the pandemic, in terms of negative and positive psychological and practical effects. By describing the experiences the HAs had through their role and what kind of challenges and coping strategies they applied, we will provide policymakers with relevant insight to consider when implementing similar measures in the future in a fair and sustainable way.

## Methods

### The HA- project

The HA-project took place in Bergen, Norway and the surrounding municipalities from spring 2021 to fall 2022. The HA-project was a collaboration between Bergen municipality, Caritas (a nongovernmental organization for migrants in Bergen), and the Pandemic Center at the University of Bergen. Caritas Bergen held the responsibility for the organization and logistics of the project, which included the recruitment of a total of 75 HAs from Poland, Somalia, Eritrea, Iraq, the Philippines, Palestine, Turkey, Ethiopia and Ukraine (from 2022). The Pandemic Centre at the University of Bergen had overall responsibility for the execution of the meetings and the academic aspects during the project. The municipality of Bergen had a coordination function towards their own sector authorities and other organizations. They also organized vaccination for undocumented migrants.

The core of the HA-project was to link the medical experts, the municipality as provider of services, and the migrants in their role as ambassadors as equal partners with different types of expertise. The primary objective for the HA- project was to improve communication with migrants in Bergen about themes related to the pandemic by giving the HAs access to timely and up-to-date information, while also updating the municipality and the academic institutions about challenges encountered in the field.

The meetings were held in Norwegian, since this was the common language for the HAs. At the beginning of each meeting, the leader of the Pandemic Center interviewed one or two medical experts, persons from the municipality or social providers based on questions that were relevant for the migrants. The focus was on establishing an open dialog by addressing specific questions gathered at community level using easy-to-follow language and showcasing that all questions were welcome and allowed. After the interviews, the HAs gathered in several different language-groups in digital rooms where they could process and discuss the information in their mother tongue. Afterwards, all participants gathered again for plenum discussion, and the HAs could ask further follow-up questions and learn from the health experts and other HAs on how to handle challenging situations. After attending meetings, the HAs employed various strategies to disseminate the information they had acquired to migrants within their local communities, using their native languages.

As both the medical and the geopolitical situations changed, there were two stages during the HA-project: during 2021 when the meetings were digital and the HAs did not receive payment for their work, and from the beginning of 2022 when Ukrainian refugees were included. The later stage also held physical meetings, and the HAs were paid a thousand NOK (approx. 100 euro) for their work.

### Participants and recruitment

The HAs were selected as migrants with key positions in their communities with the additional criterium that they had some kind of health background, but they were not acting in a professional capacity for the HA-project. Although they were initially recruited as volunteers, they were remunerated at a later stage in the project, which removes them from the categorization of “volunteer”. Nevertheless, they remain individuals with some experience in health care work, spreading health information outside of their work settings, without the initial promise of payment for their efforts.

The informants for this study were selected among the 75 HAs in the HA-project, most of them women. Recruitment took place in January 2023, during breaks and after the ‘HAs’ physical meetings´ at Caritas. During these times, the master´s student approached the HAs to explain that participants were being sought for interviews related to the master thesis evaluating the HA-project. Typically, between 15 and 25 HAs participated in the physical meetings at that stage of the project. It was emphasized that participation was voluntary for HAs and was entirely separate from the HA-project itself.

The inclusion criteria for recruiting informants were being engaged as HAs and actively taking part in the HA-project; attending meetings at the time of recruitment. We included both HAs who participated from the start of the project and those who had joined the project in 2022 to obtain a wide range of experiences and the most comprehensive variation of perspectives. An effort was made to recruit as many informants as possible, resulting in a final sample of seven informants. The primary reason why several of the HAs did not participate in the study was a lack of available time, as many were already dedicating a significant portion of their own personal time to the HA-project. However, our on-going assessment of the material concluded that the quality of the information we obtained from the HAs was of high quality, and no new themes emerged in the last interviews. Accordingly, we determined that the data acquired from the informants were adequate for describing the overall experiences of the HAs.

### Registration and consent

The project was registered at the data protection office (PVO) at the Norwegian Agency of Shared Services in Education and Research (SIKT), with the reference number 627457. All the informants received written and oral information about the purpose of conducting the interviews and their rights as participants in the study prior to the start of the interviews. We communicated to the informants that participation in the study was voluntary and they could withdraw their consent at any time, and doing so would not result in any negative consequences.

### Data and data collection

We conducted qualitative individual interviews to capture the nuances of the informants´ experiences in their role as HAs in their own communities. The interview guide (Supplementary 1) was developed by the first and last authors and commented by the rest of the authors, and included questions to explore both positive and negative experiences linked to their participation in the HA-project. More specifically, we wanted to gain knowledge on how the HAs experienced receiving and giving information, any emotions attached to the new role, the social aspects of the project and the project’s costs for the HAs. In the context of this research, the term “costs” encompasses not only the tangible financial expenditures but also factors such as the HAs´ work effort, preparatory activities, and time allocation from their personal leisure or other professional commitments.

The interviews were conducted in Bergen by the first author at a café, Caritas facility and in the psychology department at the University of Bergen. The language used in the interviews was either Norwegian or English as preferred by the informant. The interviews lasted from 15 to 40 min. The audio recordings were transcribed and anonymized, then the transcribed documents were stored in SAFE (The University of Bergen’s online data storage program for safe processing of sensitive personal data). Audio records were then deleted.

### Data analysis

Four interviews were held in English and translated to Norwegian. Data analysis was conducted in Norwegian by the first author assisted by the last author, using systematic text condensation as described by Malterud (2018), through four steps; (i) obtaining an overview of the collected material, (ii) identifying and sorting meaning units, (iii) condensing the content from the meaning units, and finally (iv) summarizing the meaning of the material to text [21].

## Results

A total of six women and one man were interviewed. Three were from Ukraine, the rest from Palestine, Iraq, Eritrea and Turkey. There was variation in age and how long the informants had lived in Norway. Four of the participants had joined the HA-project in 2022, when the meetings were physical, while three informants had been involved from the start of the project, which was organized on-line. Table 1 describes the informants.

**Table 1:** Overview informants HAs

Informant ID	Age	Gender	Country affiliation	Time spent in Norway
H1	30–40 years	Female	Ukraine	Under one year
H2	40–50 years	Male	Palestine	Several years
H3	30–40 years	Female	Ukraine	Under one year
H4	30–40 years	Female	Ukraine	Under one year
H5	50–60 years	Female	Iraq	Several years
H6	30–40 years	Female	Eritrea	Several years
H7	30–40 years	Female	Turkey	Under one year

**Fig. 1:**
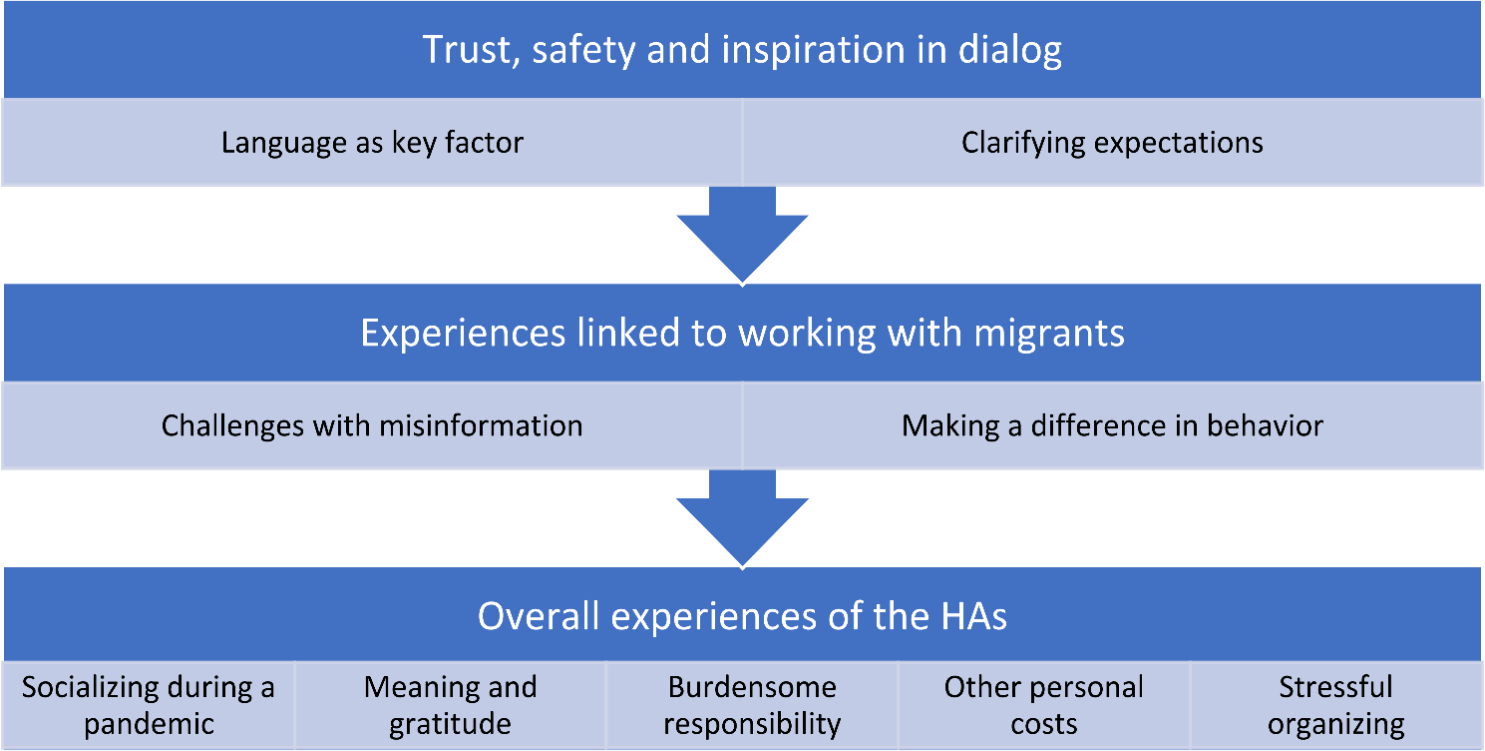
Overview main themes and subthemes from interviews with HAs

Three main themes emerged from the data: experiences linked to organization of the project, experiences linked to working with migrants, and the overall effect on the HAs themselves. Fig 1 shows an overview of these main themes and the subthemes.

### Experiences linked to the organization of the project

The organization of the meetings aimed to foster dialog among the HAs, the municipality and the health experts. The informants explained that the way of managing the language and knowledge barriers by using plain Norwegian language and allowing all kind of questions enhanced the trust, safety and cooperation throughout the project. However, HAs from Ukraine reported some caveats linked to the unclear explanation of the organization and the use of language, which they described as negative experiences.

#### Trust, safety and inspiration in dialog

Several informants expressed that the information they obtained during the meetings was trustworthy, correct and practically useful, and thus contributed to enhance their ability to answer the migrants’ questions, challenge misinformation and have confidence that the information they communicated was correct:

*“I’ve heard smart people have attended this lecture*,* so I was pretty sure this information is appropriate*,* and this information is good.” – Female*,* Ukraine*.

*“It was very positive. We have received a lot of information about the pandemic. So that was very useful information*,* I think. We were updated every time. So that was very positive.” – Female*,* Eritrea*.

Furthermore, HAs expressed safety in being able to share and discuss experiences with other HAs and together find solutions to the challenges they faced. The dialogue with health experts and the discussion they had in interaction with the other HAs strengthened them in their efforts disseminating information:

*“So they (health experts) had read a lot of research and they have also worked in the field*,* for many years. So this strengthened me. Made me more confident to give information. It was one of the things that made me more confident. Also the other thing that was that the experiences from many different HAs*,* this also made me more confident*,* since I hear from*,* for example*,* from other HAs how they solved a problem*,* so I gained experience on how we can do it.” – Male*,* Palestine*.

HAs gained new professional relationships through participation in the project. One HA described that the exchange of knowledge and experiences with other HAs inspired her:

*“(…) being there with other migrants and discussing the topic and all of us feel very*,* very inspired” - Female*,* Ukraine*.

#### Language as a key factor

The use of Norwegian as the common language at the meetings generally worked well, and the HAs who participated from the start of the project had no objection to this. The language groups where they spoke their native language gave additional support, as the HAs could clarify Norwegian terms, learn from the other HAs and discuss the themes from the interviews, preparing them to disseminate information to migrants in their native language.

However, Ukrainian HAs, who became involved in the HA-project in 2022 shortly after their migration to Norway, believed that the meetings would be available to them in English. One of the Ukrainian HAs was particularly affected by this, as she felt responsibile for the whole group of Ukrainian HAs. Upon arriving at the meeting, this HA was discouraged from learning that the meeting was held primarily in Norwegian. This was experienced even worse, as this participant was unexpectedly assigned the role as interpreter during the meetings as an ad hoc solution when the challenge became obvious for the organizers during one of the meetings:

*“I tried and I failed because it was such a speed (…)*,* it was giving us so much information without being considerate of whether people understood it or not”* - *Female*,* Ukraine*.

#### Clarifying expectations

In addition to language, a clear difference emerged in interviews with HAs that participated from the start of the project and those who were included later regarding how much clarity they had in what their role entailed. Those who were included early had a clearer understanding of their role as HAs; that their main task was to convey information about the pandemic to migrants. HAs included later expressed that they were unaware that the project´s intent was to disseminate the information they received to other migrants. Instead, they had understood the purpose to be primarily to receive information from the health experts. When the intent was finally clarified to the Ukrainian HAs, it was at the end of the project and the information was not as relevant to spread to migrants in their local communities. The Ukrainian HAs therefore expressed in retrospect that it would have been beneficial to receive information about the intent earlier so that they could have fulfilled their duties as HAs. A lack of clarity regarding expectations was expressed as a negative experience among the latter group:

*“Maybe spread more information. Because I spread only in my group*,* which I was comfortable with (…) everyone was from Ukraine. (…) good if I could help more people.” - Female*,* Ukraine*.

*“(…) I didn’t know that I had to spread this information to other people” - Female*,* Ukraine*.

Furthermore, the HA-project intended to evaluate how the project benefitted in terms of fighting misinformation encountered in the communities among the HAs themselves. For this purpose, questionnaires with simple wording were handed out by the organizers at the beginning and the end of the meetings, checking for misinformation and to test the learning effect of the meetings on the HAs themselves. This was explained at the beginning of the project to all the HAs. However, the informants reported that this had not been properly explained to the HAs who entered the project later. Thus, several HAs who joined in 2022 found the design of the questionnaires problematic. Some HAs perceived the questions as condescending due to its simple wording, but other Ukrainian HAs who did not understand written and spoken Norwegian medical terminology very well at that time found it challenging to answer:

*“To be honest*,* it’s one thing I don’t like. (…) the questions in the pre and post questionnaire (…) There were some stupid questions (…) you know*,* I’m a well-educated person*,* I have two master’s degrees and being an immigrant doesn’t mean you don’t know anything. (…) I did not like those questions to be honest with you (…) I felt that*,* you know that thinking that migrants all come from always eh a negative background or are illiterate.” - Female*,* Turkey*.

*“(…) I tried to translate it for the Ukrainians*,* but also for myself despite the fact that I am at B1 level in Norwegian. I would appreciate it if this information was also in a copy in English” - Female*,* Ukraine*.

### Experiences linked to working with migrants

In addition to the organization of the project, the HAs spoke about experiences and emotions linked to their role in interaction with migrants. Several HAs expressed certain challenges with disseminating health information, but also positive emotional effects as a result of being put in a position to help and make a difference for some migrants.

#### Challenges fighting misinformation

Several HAs described debunking misinformation and what they considered “conspiracy theories”, especially about vaccination, as the most challenging task in their interactions with migrants. The HAs indicated that this difficulty stemmed from the migrants in the community being exposed to diverse and conflicting information from various media sources, which could create a sense of uncertainty and hesitation when they were subsequently presented with accurate information from the HAs:

*“It wasn’t so easy (…) how we solved this wasn’t so easy. We also noticed that people didn’t believe us very often*,* since they read about things every day(…) Also someone else said to me* <*Yes*,* but this was a lie also the drug factories are supposed to sell new drugs so that’s why they did it*>. *So there are different things*,* also it comes on the Facebook pages or other social media*,* TikTiok that supports those things*,* yes. I think the biggest challenge was with social media.” - Male*,* Palestine*.

*“There were different opinions. Some were just interested in the topic. Someone said < I don’t believe it. It’s not a disease*,* it’s something people made up>*,* you know*,* these conspiracy theories.” - Female*,* Ukraine*.

#### Making a difference in behavior

Getting migrants tested or vaccinated because of receiving correct health information about the pandemic was experienced by several HAs as the most important achievement during the project:

*“The biggest one I’ve done is the assignment that was at Nygård school (…) maybe 35 or 40 people in three groups who spoke Arabic and*,* the last group maybe spoke Kurdish*,* but they understood Arabic. (…) a lot of questions (…) agreed to get vaccines after this assignment.” - Male*,* Palestine*.

*“As for the vaccine; it was not easy that we can talk and let people agree with us that they should take the vaccine*,* but after that meeting*,* and what we felt was important*,* they said < we will go and take the vaccine > after we had spoken with them. Because several of them or many of them… most of them heard from their home country that < that vaccine doesn’t work*,* it’s not good to take it*,* maybe people die too>” (…) - Female*,* Iraq*.

### Overall experiences of the HAs

The HAs reported predominantly positive experiences by taking part in the project through socializing with peers with different backgrounds, and by being recognsied as key contributors in addressing a health crisis. Nevertheless, there were some burdensome consequences linked to their participation, primarily relating to the sense of responsibility they felt both in terms of delivering correct information and engaging personally in the role. In addition, some experienced a clash in expectations related to their roles that affected them in a negative way.

#### Socializing during a pandemic

The participants described positive social and professional aspects by being able to socialize with other HAs during the pandemic. An informant highlighted that migrants rarely interact with people from different cultural backgrounds than themselves. Several HAs made friends through the project:

*“We don’t have many friends here because we settled here recently (…)I think it was a social thing (…) two new friends (laughter) that I got from the meetings.”* - Female, Turkey.

#### Meaning and gratitude

All HAs expresses joy, meaning and gratitude for having the opportunity to participate in the project and contribute during a crisis. This was true even for the HAs who joined the project in 2022. The informants described how migrants often feel underprivileged and put in positions to ask questions rather than answering them. Through their role as HAs, several HAs experienced being seen as key actors by experts and the municipality and as a resource for others. Furthermore, it felt good to be able to contribute with necessary health information to migrants within their immediate environment and support them in making informed decicions during a crisis:

*“You’re here in Norway as an migrant (…) you don’t feel so privileged (…) then you get this responsibility and now you say < wow*,* I have a purpose > It’s very nice. <Oh I know something>*,* you know < I’m the person who (laughs) gives the information> (…) I can teach people something which is nice*,* because normally as an migrant you always ask questions because you don’t know how the system works (…) so it was good for me.” -Female*,* Turkey*.

Despite the challenges the Ukrainian HAs faced, they still expressed gratitude for being able to take part in the project. They accredited this to the HA-project being the best of the few options available for the Ukrainian HAs at that time to obtain quality information about pandemic related issues, which contributed to their positive evaluation of their experience. Despite the limiting factor of not understanding every word said at the meetings, they still had many opportunities to discuss amongst themselves in their language group, socialize with other HAs and ask questions to health experts.

*“It was wonderful to get information from the health experts. (…) We had very nice conversations with the other migrants. We came to the meetings because we are interested in the subject and we had good conversations during the meetings and after the meetings there was a very nice atmosphere and a really good opportunity to meet and talk with other people.” - Female*,* Ukraine*.

#### Burdensome responsibility

Despite the HAs positive experiences, having the responsibility of communicating information and fighting misinformation in their community was sometimes described as a challenge. The HAs coped with these challenges by seeking support and solutions from other colleagues, HAs and the health experts:

*“It was not that simple. Eh it was positive. I think it was fine. And for me to get a lot of information*,* it’s a lot of responsibility too. Eh*,* I was afraid of giving the wrong answers. So I was very careful*,* and those questions that were the most difficult*,* I discussed them with my colleague who has worked during the pandemic at a hospital in Bergen. So she had a lot of information as well. She was a nurse. (…) Although we are from the same country*,* I have not met her. Then we got to know each other more and we became friends as colleagues. We had a lot of plans*,* what to do*,* how to handle*,* how to fix and how to organize. So she had a big network and I had a big network. So it was much easier.”- Female*,* Eritrea*.

*“There were a lot of challenges with it. It wasn’t like that…What can I say…maybe not just talking to people that were challenging*,* there were other things that apply to us as ambassadors that we were very concerned about*,* we had our own lives. So this was another challenge. (…) it’s not so easy to get into other people’s lives (…)* - *Male*,* Palestine*.

#### Other personal costs

During the interview process, eliciting conversations about the cost or additional efforts incurred by HAs in fulfilling their project-related responsibilities posed a challenge. A subset of HAs voluntarily dedicated their free time to assisting individuals who were unwell, thereby demonstrating a strong sense of moral obligation and finding personal fulfillment in these acts. Consequently, certain HAs undoubtedly bore not only time but also economic costs related to meal preparations and food expenses. They engaged in activities such as delivering meals to infected migrants, making late-night phone calls, and assisting with grocery shopping for affected individuals. Interestingly, most HAs hesitated to discuss economic costs within the context of their altruistic endeavors, instead emphasizing their sense of responsibility toward those they aided:

*“I had to buy food to deliver eh to the door. (…)I have bought everything she needs(…)twice I have cooked food myself at home then delivered it to her. (…) she is an elderly lady so she needs help*,* I had to do it. (…)our culture to help others and give food(…) So I don’t think like that about finances…” - Female*,* Eritrea*.

As a follow-up question to this quote, the informant was asked whether they always had this work effort and sense of responsibility or whether this was especially for the HA-project. The answer from the informant was descriptive of several HAs sense of responsibility:

*“I’m like that truly. People tell me < you work so hard!> I forget to take a break. I like to help people. I am efficient. I also spend my free time helping people. I help people go to the doctor. I help. I don’t want to disappoint people. A bit cultural*,* my background.” - Female*,* Eritrea*.

#### Stressful organizing

The HAs who joined the project at a later stage did not receive the adequate facilitations to be able to follow the project as intended. Therefore, the negative sides of the project related to unmet expectations were experienced as more stressful by this group of HAs. One informant describes some of the added burden this caused:

*“Wow okay*,* I have to work here! (…) I wasn’t sure if I wanted to be there because I had to go on my vacation*,* but then I understood that I have to be there*,* because if not they can’t be there*,* they (the other Ukrainian HAs) would not have understood anything because it would have been in Norwegian. So that’s why I postponed my vacation. But if I didn’t want to be paid a thousand NOK*,* I wouldn’t participate because you see the conditions.” - Female*,* Ukraine*.

## Discussion

Volunteering migrants participating in the HA-project and disseminating information to other migrants in their immediate environment during the pandemic experienced a combination of positive psychological states such as meaning, gratitude and resilience. However, their role as HAs also posed some burden in terms of psychological stress.

Almost all HAs highlighted the quality of the information they obtained from the health experts as a contributor to their positive experience and sense of security through the project. The dialogue and the exchange of knowledge with other HAs in the meetings were additional factors enforcing their sense of security. The negative experiences, especially among the Ukrainian HAs, were linked to the unclear communication of the organization of the project, seemingly through different expectations regarding language and organization of the meetings. Despite this, the Ukrainian HAs expressed gratitude for being included in the project; to obtain and discuss with peers, receive quality information, socialize and to have the opportunity ask questions to health experts.

Coping is about using cognitive and behavioral strategies to handle challenging situations [20]. Focusing on gratitude can be one strategy to be able to cope in stressful situations [23]. Socialization with other HAs and the dialog in the meetings had positive effects; strengthening and inspiring the HAs through the challenges encountered in their work. Furthermore, although deconstructing misinformation among migrants was a challenge for several HAs, they overcame it through conversation with each other at the meetings. Hence, our results confirm the findings on coping by Folkman (1997) showing that even during challenging periods, individuals can experience positive affective states [22] through coping strategies such as engaing in discussions within safe and trusted environments with the support of experts [23], which appear to have had a stress-reducing effect for the HAs through the project.

Seeking meaning has also been described as a way of copying (24–25) that seems to apply to our informants. A study conducted during the COVID-19 pandemic showed that despite stress, depression and anxiety among health workers on the front line, nearly two thirds found greater meaning and purpose in their lives [25]. In a study including both health care workers and volunteers during the pandemic, volunteers had higher levels of psychological stress but also higher levels of happiness compared to those who did not volunteer during the pandemic [19]. In our study, many of the informants seem to have sought meaning by getting an overview of what the problem was, namely, misinformation. This is supported by research on meaning and resilience; showing that by obtaining an overview of what the challenges are and by having a problem-solving attitude, one can clarify what is important to work towards and therefore experience meaning in the work one is preforming (23–24).

Although the HAs agreed that the project overall was a positive experience, mismatch of expectations and language challenges for Ukrainian HAs were the main causes of negative experiences and frustration reported. Encounters with healthcare services for migrants can also be influenced by a lack of interpretation services and language challenges [18, 26]. It might be concerning that even a project meant to provide adapted information about health directed at migrants was not able to provide adequate information and have a common agreement of expectations with all the participants. However, this can also be considered as one of the consequences of the rapid transfer of responsibility to NGOs during the pandemic, with limited time to adapt to changing conditions, such as the arrival of Ukrainian refugees.

The experience of being needed to help the government to reach out with health information to migrants was an additional source of positive experiences for the HAs. In contrast to the discourse of portraying migrants as problems in the midst of a pandemic, HAs expressed pride in demonstrating that they possessed distinctive resources; both in their health background and with their migrant background, which enabled effective communication with the migrants and understanding of what migrants went through and lacked [5, 18].

An unanticipated finding in our study-likely related to seeking meaning-was that the HAs sense of responsibility extended beyond the description of their designated roles as HAs, helping migrants in practical ways and hesitating to discuss financial or personal costs in the context of these acts. Importantly, HAs are resourceful individuals who had several areas of responsibility outside of their role as HAs. Their sense of responsibility stemming from their other roles held outside their function as HAs, may have contributed to their motivation to exceed the expectations set by the HA-project organizers. Furthermore, the HAs expressed a concern for who would help the migrants if they did not do it. This raises the question of the ethical grounds of outsourcing crisis-related support responsibilities to individuals who are themselves part of the vulnerable communities they are expected to assist. 

### Strengths and limitations

The HAs experiences can provide insight for policymakers on volunteer initiatives targeting migrants. Understanding challenges and coping strategies can enhance volunteer effectiveness and improve outcomes for the communities they target health initiatives to. The seven informants in our study had extensive experience in the project and were able to provide detailed information, describing the experiences of the HAs during their participation in the HA-project. Our study has both strengths and limitations related to recruitment. Informants from several migrant communities were successfully recruited, to insure the information obtained from the participants would provide a truthful reflection of the general experience of the majority of HAs. However, the study could have benefited from a lager sample and broader diversity in migrant backgrounds of the informants. This could have improved the validity of the data. Further, our sample lacked a variation in gender, as most HAs were women. Thus, it’s difficult to generalize the results for males in similar roles. Another limitation is that participants did not get a opportunity to view the findings and give feedback on the data we acquired from them.

Despite the limitation of a small sample and lack of variations in genders, the quality of the information we got from the HAs was rich and new themes did not appear in the last interviews. We describe strategies the HAs used to cope with challenges, in particular in their clear expressions of gratitude to be set in a position to help others, finding meaning in their tasks and collaborating in a trusted environment. Our analysis, however, cannot draw conclusions as to whether these strategies were consciously implemented by the HAs to cope with the challenges they encountered, emerged as part of subconscious behavior, or resulted from other underlying factors. 

## Conclusion

The experiences of the HAs can provide valuable insight for policymakers regarding the considerations necessary when developing initiatives that utilize volunteers to reach out to migrants during a crisis. Clarifying the roles of the volunteers and creating safe environments with easy access to health care experts and possibilities of discussing strategies with peers, may be beneficial in securing resourceful individuals health and effectiveness while also enhancing the positive impact of their work on the individuals or communities they serve.

## Electronic supplementary material

Below is the link to the electronic supplementary material.

Supplementary Material 1

## Data Availability

The data that support the findings of this study are available from the corresponding author on reasonable request.

## Declarations

The content of the manuscript has not been published or submitted for publication elsewhere. There are no issues relating to journal policies. All authors have read and approved the manuscript for submission.
