# Supplementary material for: Migrants’ experiences as health ambassadors during the COVID-19 pandemic in Norway-a qualitative study
Source: Int J Equity Health. 2025 Apr 30;24:118. doi: 10.1186/s12939-025-02480-9 (PMC12042631; doi:10.1186/s12939-025-02480-9)
Supplement: Supplementary file 1 — Supplementary Material 1 [file 12939_2025_2480_MOESM1_ESM.docx]

**Supplementary 1: Interview guide**

Thank you very much for having the opportunity to participate!

The interviewer hands out a consent form and explains what the purpose of the project is, what the topics of the questions will be and what we will use the interview for. Informs about anonymity.

Duration: 30-60 min

Do you have any questions before we start?

Age:

Number of years in Norway:

Nationality:

Norwegian understanding:

1. How were you contacted with the request to become a health ambassador?

2. How did it feel to get information and answers to questions about the pandemic from health experts?

3. In what way did you feel that your questions about the pandemic were adequately answered and discussed?

4. How did you spread the information to others?

5. In what way did you feel that you were able to answer the questions the migrants had?

6. In what way did you experience the migrants responding to receiving information from you about the pandemic? (Do you know, for example, if someone went to get tested after you talked to them about this or if they got vaccinated after you talked about vaccination?)

7. How did it feel to have a responsibility to provide health information to migrants during the pandemic?

8. What has been stressful in the role as health ambassador?

9. What has been positive in the role as a health ambassador?

10. In what way could participation in the project be experienced socially during a period it was, with many shutdowns and when many were alone a lot?

11. Has being a health ambassador taken time away from your usual work?

- What consequences has this had for you?

12. Has the role of health ambassador taken more time from your free time than you wanted?

- What consequences has this had for you?

13. In your role as a health ambassador, did you have to carry out tasks that you had not imagined in advance?

- In what way?

14. Have you paid for anything or spent money in other ways in connection with your role

as a health ambassador?

15. What would you, as a health ambassador, wish was done differently in a similar,

future project?

Is there anything else you would like to say or add? Can we contact you again if the need arises?

Thank you very much for your time!
